# Breast cancer with choriocarcinomatous and neuroendocrine features

**DOI:** 10.1590/S1516-31802001000400009

**Published:** 2001-07-07

**Authors:** Osvaldo Giannotti, Luciana Nakao Odashiro Miiji, Marta Vainchenker, Ângela Navarro Gordan

**Keywords:** Breast cancer, Choriocarcinomatous features, Choriocarcinomatous and neuroendocrine features, Câncer de mama, Câncer com características coriocarcinomatosas, Câncer de mama com características coriocarcinomatosas e neuroendócrinas

## Abstract

**CONTEXT:**

Breast cancer may express the presence of b-human chorionic gonadotrophin in 12% to 18% of cases, using immunohistochemical reactions. Usually the tumors will show positivity in a few scattered cells. Breast cancer with choriocarcinomatous features, as reported by Saigo and Rosen, is a distinct variant of breast cancer. We report a case of breast cancer with choriocarcinomatous and neuroendocrine features.

**OBJECTIVE:**

Thisis a case report of an invasive ductal carcinoma of the breast with choriocarcinomatous and neuroendocrine features.

**DESIGN:**

Case Report.

**CASE REPORT:**

A 50-year-old Brazilian woman underwent surgery for a lump in the right breast, which had been first noticed about 3 months earlier. The surgery consisted of quadrantectomy followed by right mastectomy with ipsilateral axillary lymph node dissection. The specimen from the quadrantectomy revealed a 7 × 6.5 × 4.5 cm tumor. Histology of the lesion showed the presence of an invasive ductal carcinoma with areas of giant cells and intense atypia. The immunohistochemistry was positive in the pleomorphic areas for human chorionic gonadotrophin, while the less pleomorphic areas showed positivity for synaptophysin and chromogranin.

## RESUMO

**CONTEXTO:**

O câncer de mama pode expressar a presença da gonadotrofina coriônica humana-beta (b-HCG) em 12% a 18%, por reações imunohistoquímicas. Usualmente, apenas algumas células do tumor mostrarão positividade. Câncer de mama com características coriocarcinomatosas, descrito por Saigon e Rosen, é uma variante distinta do câncer de mama. Descrevemos um caso de câncer de mama com padrões coriocarcinomatosos e neuroendócrinos.

**OBJETIVO:**

Esse é um relato de caso de um carcinoma ductal invasivo de mama com características coriocarcinomatosas e neuroendócrinas.

**RELATO DE CASO:**

Mulher de 50 anos de idade, brasileira, apresentou um nódulo na mama direita que foi notado primeiramente três meses antes da cirurgia, fez uma quadrantectomia seguido de uma mastectomia direita com linfadenectomia ipsilateral. A peça cirúrgica da quadrantectomia mostrou um tumor de 7 × 6, 5 × 4, 5 cm. A histologia da lesão revelou um carcinoma ductal invasivo com áreas de células gigantes e intensas atipias. O estudo imunohistoquímico revelou positividade nas áreas pleomórficas para gonadotrofina coriônica humana (b-HCG), enquanto que as áreas de menor pleomorfismo mostraram positividade para sinaptofisina e cromogranina.

## INTRODUCTION

Breast cancer may express the presence of b-human chorionic gonadotrophin in 12%^1^ to 18% of cases,^2^ using immunohisto-chemical reactions. Usually the tumors will show positivity in a few scattered cells. Of women with breast cancer, 12 to 13% show elevated levels of serum human chorionic gonadotrophin.^2^ Breast cancer with choriocarcinomatous features, as reported by Saigo and Rosen,^2^ is a distinct variant of breast cancer. Histologically, such tumors show pleomorphic areas of large-sized, oval-shaped tumor cells with a high nuclear-to-cytoplasmic ratio and increased nuclear chromatin. The cytoplasm of the tumor cells is pale to eosinophilic with occasional periodic acid-Schiff stain-positive intracytoplasmic eosinophilic globules. Occasional multinucleated giant cells are also seen. These findings are similar to choriocarcinoma originating from the genital tract.^3^ We report a case of breast cancer with choriocarcinomatous and neuroendocrine features.

## CASE REPORT

### Clinical Course

The patient was a 50-year-old Brazilian woman who reported a mammary lump with progressive growth in the upper outer quadrant of the right breast. It was first noticed about 3 months before she underwent quadrantectomy. The specimen revealed a 7 × 6.5 × 4.5 cm tumor with infiltrative borders and areas of necrosis and hemorrhage of the cut surface. Histology of the lesion showed the presence of an invasive ductal carcinoma with areas of giant cells and intense atypia.

Following confirmation of a malignant breast tumor, a right mastectomy with ipsilateral axillary lymph node dissection was performed. During the operation, no distant metastasis was observed in any organ. Measurements of serum human chorionic gonadotrophin and female sex hormones were not performed in the preoperative period.

### Pathologic findings

The specimen showed an ill-defined tumor, 7 × 6.5 × 4.5 cm in size, located in the upper outer quadrant of the right breast with infiltrative borders. The cut surface showed areas of necrosis and hemorrhage.

**Figure 1:**
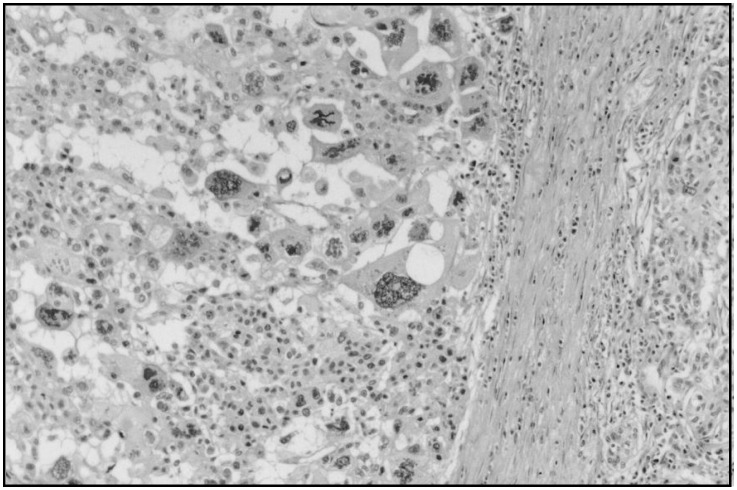
Pleomorphic area with large-sized, atypical tumor cells, multinucleated giant cells and atypical mitosis (HE, X100).

Histologically, the tumor revealed an invasive ductal carcinoma with pleomorphic areas that showed multinucleated giant cells and intense atypia and areas with less pleomorphism (Figure 1).

Vascular permeation of tumor cells was inconspicuous. The product from the right mastectomy showed no residual neoplasia. There was also no angiovascular, skin or nipple infiltration. All of the 20 axillary lymph nodes dissected were free from metastasis.

### Immunohistochemical findings

The tissue for immunohistochemical investigation was embedded in paraffin after being fixed in 10% formalin solution. The specimens were stained with chromogranin, synaptophysin, human chorionic gonadotrophin and cytokeratin (AE-1/AE-3) using the avidin-biotin peroxidase complex method with hematoxylin counterstaining.

**Figure 2:**
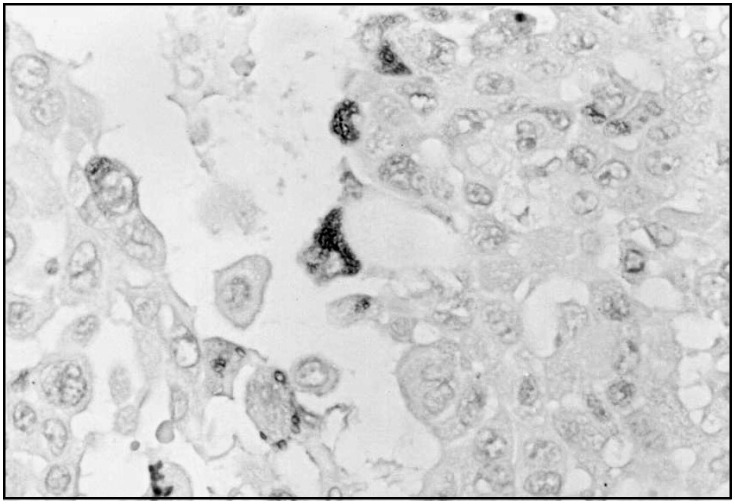
Immunohistochemistry: large and bizarre tumor cells showing positivity for chorionic gonadotrophin (X400).

The reaction was positive in the pleomorphic areas for human chorionic gonadotrophin (Figure 2), while the areas with less pleomorphism showed focal positivity for synaptophysin and chromogranin. The cytokeratin (AE-1/AE-3) was positive as expected and the other immunohistochemical findings were focal positivity for estrogen receptor and negativity for progesterone receptor.

## DISCUSSION

Some mammary carcinomas are able to synthesize hormones not considered to be normal products of the breast. Such tumors have been found to produce peptide hormones including human chorionic gonadotrophin, calcitonin and epinephrine. Breast cancer may express the presence of b-human chorionic gonadotrophin in 12%^1^ to 18%^2^ of the cases, using immunohistochemical reactions. Usually the tumors will show positivity in a few scattered cells. In our case, b-human chorionic gonadotrophin was focally positive. Another unexpected finding was the positive reactivity in the less pleomorphic areas to both chromogranin and synaptophysin, showing neuroendocrine differentiation in addition to the choriocarcinomatous features.

Neoplasms with neuroendocrine differentiation do not constitute a specific histopathological category of female mammary carcinoma, but it is apparent that there is a group of mammary carcinomas capable of producing ectopic hormonal substances. The recognition of these features is necessary for defining their clinical characteristics. Moreover, some studies show that nearly half of the patients had axillary nodal metastasis and also a higher frequency of recurrence and death due to breast carcinoma. However, there have been no case-controlled studies of patients who have mammary carcinoma with neuroendocrine differentiation with age, which compare these with stage-matched control groups. Thus, neither ectopic hormone production nor endocrine differentiation has proven to be a factor that critically influences prognosis or treatment.

On the other hand, the choriocarcinomatous features of this case allow it to be classified as the distinct variant of breast cancer reported by Saigo and Rosen.^2^ It is a unique variant of ductal carcinoma with focal choriocarcinomatous components. The choriocarcinomatous component is usually associated with conventional infiltrating ductal carcinoma such as solid tubular carcinoma.^3^ Mucoid carcinoma of the breast with choriocarcinomatous components in the metaplastic area has also been reported.^1^ The cellular origin of breast cancer with choriocarcinomatous features is uncertain. Nevertheless, conventional ductal carcinomas in breast cells may sometimes show positive immunohistochemical reactivity for human chorionic gonadotrophin.^3^

Finally, the importance of diagnosing the choriocarcinomatous features in an invasive ductal carcinoma is that such tumors are highly malignant. Their clinical course is usually aggressive and most patients die within a few months due to multiple metastases.
